# Small-Sample Estimation of the Mutational Support and Distribution of SARS-CoV-2

**DOI:** 10.1109/TCBB.2022.3165395

**Published:** 2022-04-06

**Authors:** Vishal Rana, Eli Chien, Jianhao Peng, Olgica Milenkovic

**Affiliations:** Department of Electrical and Computer EngineeringUniversity of Illinois Urbana-Champaign 14589 Champaign IL 61801 USA

**Keywords:** Small-sample support estimation, small-sample distribution estimation, Chebyshev and weighted Chebyshev approximations, Good-Turing estimators, virology

## Abstract

We consider the problem of determining the *mutational support and distribution* of the SARS-CoV-2 viral genome in the small-sample regime. The mutational support refers to the unknown number of sites that may eventually mutate in the SARS-CoV-2 genome while mutational distribution refers to the distribution of point mutations in the viral genome across a population. The mutational support may be used to assess the virulence of the virus and guide primer selection for real-time RT-PCR testing. Estimating the distribution of mutations in the genome of different subpopulations while accounting for the unseen may also aid in discovering new variants. To estimate the mutational support in the small-sample regime, we use GISAID sequencing data and our state-of-the-art polynomial estimation techniques based on new weighted and regularized Chebyshev approximation methods. For distribution estimation, we adapt the well-known Good-Turing estimator. Our analysis reveals several findings: First, the mutational supports exhibit significant differences in the ORF6 and ORF7a regions (older versus younger patients), ORF1b and ORF10 regions (females versus males) and in almost all ORFs (Asia/Europe/North America). Second, even though the N region of SARS-CoV-2 has a predicted $10\%$10% mutational support, mutations fall outside of the primer regions recommended by the CDC.

## 1 Introduction

Viruses mutate due to unreliable replication of their genetic content and their need to evolve, adapt and evade the immune system of the host organism. As viruses accumulate mutations, some of them may become advantageous to the survival of the virus and help it circulate more widely in the host population. Simultaneously, the more the virus circulates in a population, the more likely it mutates leading to new variants that cause even more aggressive host invasion and spreading. As a result, the rate of mutation varies widely across viral families [1] and mutational and fitness landscapes of viruses are frequently used to assess their potential to spread within diverse subpopulations and communities [2], [3], [4].

The definition of a viral “mutation rate” varies significantly [1], [5]. What is referred to as the *genomic mutation rate* is the product of the per-nucleotide site mutation rate and the genome size, and it represents the average number of mutations each viral offspring has with respect to the parental (or ancestral) genome. RNA viruses have a per site mutation rate that lies in the range $10^{-6}-10^{-4}$10-6-10-4 per nucleotide site per cell infection [5]. The mutation rate of a virus is also often equated with the rate at which errors are made during replication of the viral genome. Nevertheless, it is clear that replication errors are not the only source of viral mutations. Hence, some other estimates are based on counting the mutations in sequenced genomes, using a reference corresponding either to Patient 0 (the first infected individual) or more frequently, to Patient 1 (the first individual that was sequenced). In the former context, the genome mutation rate for SARS-CoV-2 is estimated to be 2-3 mutations a month [6]. Defining the genomic mutation rate of a population is an even more challenging task as hosts may harbor viruses with widely different mutation rates.

We define the mutational support of a virus as follows. First, we declare the viral genome of Patient 1 or some other patient as a reference and index all locations along the genome. The mutational support set of a single viral genomic sequence equals the set of locations where it disagrees with the reference. Therefore, the mutational support set cardinality equals the Hamming distance between the reference and the sequence under consideration. The *mutational support of a population (henceforth, mutational support)* of viral genomes equals the size of the union of the individual mutational support sets. It is impossible to directly observe the mutational support of a population as not all patient's viral genomes are sequenced and as the mutations change in time. To estimate the mutational support, one can use a limited number of samples and count the total number of genetic sites mutated in at least one viral genome encountered in the host population. Counts (or maximum likelihood (ML) estimators) only offer good estimates of the actual mutational support when the number of genomic samples is significantly larger than the length of the viral genome. In other words, simple counting of mutations when only a small number of sequenced genomes are available may produce inaccurate estimates due to unseen mutations (caused by not having sequenced every individual and by not being able to account for all circulating mutations). The small-sample effect is a well-known phenomena extensively studied in the machine learning community [7], [8]. Nevertheless, to the best of the authors knowledge, the problems of mutational support and mutational distribution estimation in the small-sample regime have not been addressed in the literature. We argue that this problem is of significant relevance as its successful solution may be used to assess the virulence of the virus, guide primer selection for real-time RT-PCR tests during the early stages of an outbreak and help identify emerging variants [9], [10].

It has already been observed that mutations in the viral genome can be influenced by the characteristics and features of the specific host. For example, the mutation 27964C $>$> T-(S24L) in the ORF8 region is known to be more dominant in female patients than their male counterparts [11]. The expression of the angiotensin-converting enzyme-2 (ACE-2), a receptor for coronavirus [12], is observed to be higher in male patients and is a potential cause for sex-based differences in the severity of SARS-CoV-2 [13]. Immunological and hormonal differences also contribute to males having poorer prognosis [14]. There are other well-established examples of how virus evolution depends on host characteristics that go beyond coronaviruses, like the interdependence of the Human Immunodeficiency Virus (HIV) evolution and the Human Leukocyte Antigen (HLA) type of the host [15]. Even though HIV mutates significantly faster than coronaviruses, the evidence suggests that such host-dependent differences may exist in many viruses. This motivates an in-depth analysis of the mutational landscape of various human subpopulations to identify “characteristic” mutations.

Our contributions are two-fold. First, we present new machine learning methods for determining the unknown support of mutations and their distributions given sequencing data from a limited number of Covid-19 patients. The methods use efficient polynomial class estimators and exhibit state-of-the art performance on synthetic datasets. The actual genomic datasets are retrieved from the Global Influenza Surveillance Aid (GISAID) repository during the early stages of the Covid-19 outbreak. In the first step of our analysis, we use roughly 9,000 samples, which is a significantly smaller number than the length of the SARS-CoV-2 genome which roughly equals 30,000. The approach is based on weighted Chebyshev polynomial estimators and adapted Good-Turing distribution estimators, and its accuracy is evaluated based on larger sample set sizes retrieved on later dates. *We emphasize that our problem lies the so-called small-sample regime. The term small-sample regime in our setting does not pertain to the number of samples (or patients) compared to the total population of the world (or the actual number of infected individuals), despite this clearly being the case. Instead, our notion of small-sample estimate refers to the number of mutations observed within the union of all genomes (samples) compared to entire set of genomic locations.*

Second, the mutational supports are estimated for three different population types, namely according to geographic region (Asia (A), Europe (E), North America (NA)), sex (female (F)/male (M)) and age ($< 55$<55, $>55$>55). For European samples retrieved at a later time stage, estimates for females of age $< 55$<55 versus males of age $>55$>55 were analyzed as well. The estimates are used to predict mutational hotspots and compare the genomic loci with highest mutation frequency in different subpopulations. For the latter task, we further process the results by using the Jaccard distance as well as the symmetric Kullback-Leibler divergence. To determine if the mutation rates are appropriately low in the primer regions for polymerase chain reactions (RT-PCR) [16], we also examine the N ORF of SARS-CoV-2.

Our analysis reveals several important biological findings. The predicted mutational supports exhibit significant differences in the ORF6 and ORF7a regions in older versus younger patients, ORF1b and ORF10 regions in females versus males. The mutational support of the ORF1b region for young females is almost twice that of old males, while old males have a substantially larger mutational support for gene ORF10. Given that young females are much less likely to develop severe symptoms than old males, the identified potential high-mutation regions may be further examined to identify their potential role in the spread and severity/potency of the virus. Furthermore, it is important to observe that the variance of the support is extremely high in the ORF8 region, close to 200 times higher for patients above 55 years of age compared to patients below 55 years of age. Less surprisingly, there also exist statistically significant differences in the ORFs of Asian versus European and NA samples in the ORF1a,b and other ORFs. Second, despite the fact that we predict that the N region of SARS-CoV-2 will have a very large mutational support, almost all high-probability mutations fall outside of the two regions of paired primers recommended by the CDC.

It is important to point out that potential sampling biases could have an impact on our findings. GISAID data does not provide information regarding the severity of the disease; however, data deposited at this repository is not exclusively contributed by hospitals so there is no a priori reason to believe that sampling biases exist. Furthermore, note that the observed frequencies of mutations across viral genomic sites are a function of numerous and complex factors that are still poorly understood; they include population dynamics, viral-host interactions and natural selection. They also reflect the random timings at which mutations occur. As one can expect, given the lack of adequate models, our analysis and synthetic data simulations cannot completely account for the above described phenomena. Nevertheless, our methods for sequence analysis have provable guarantees for some simplified models, which is seldom the case for computational biology methods. Furthermore, the random mutation process may be nonstationary, although at small time scales one does not expect the distribution to change in a significant manner; to account for this issue, we address the dynamics of the mutation process by sampling genomes made available at different times and comparing the prediction results based on earlier (and smaller) time-stamped collections with those actually observed at a latter time. In all our experiments, we randomly subsampled the different subpopulations to the same final sample set size. Random subsampling may potentially reduce unknown sampling biases. It is worth noting that the sampling biases may persist even when a large number of samples is available, and large datasets need to be downsampled to make computations tractable [17], [18], [19]. A theoretical analysis of small-sample support estimation in the presence of sampling biases is available at [20].

The paper is organized as follows. In the Methods Section, we describe the data acquisition process, the pre-processing tasks as well as our new small-sample support and distribution estimation algorithms. The most relevant results are presented in the Results and Discussion Section.

## 2 Methods

### 2.1 Organization of the SARS-CoV-2 Genome

Typically, coronaviruses have genomes including at least six open reading frames (ORFs) [21] and [22]. ORF1a and ORF1b constitute the longest component of the genomes and are responsible for encoding two polypeptides, pp1a and pp1ab, which are jointly used to create a family of nsp proteins. This family of polypeptides includes replicase-transcriptase proteins, responsible for promoting cellular mRNA degradation and blocking the translation process in host cells, thereby impairing the operation of the immune response and proofreading. The pp1a/b polypeptides are functionally combined using proteases, such as the native chymotrypsin-like protease. Viral structural proteins are encoded by the sgRNA region, and include the ORF2 or spike (S), ORF5 or membrane (M), ORF4 or envelope (E), and nucleocapsid (N) proteins, as well proteins encoded by the ORF10 sequence. ORF3a encodes a membrane protein that interacts with proteins encoded by ORFs M, S and E and is believed to play an important role in viral release and the generation of cytokine storm; on the other hand, ORF3b encodes proteins that block the induction of interferons with antiviral activity. The ORF6 products are important virulence factors that enable the virus to escape detection by the immune system of the host.

For real time RT-PCR testing and detection of Covid-19, the oligonucleotide primers and probes are selected from the nucleocapsid (N) gene region (per CDC guidelines for the United States [23]). Countries like Germany and China have adopted primers from other genomic regions [16]. For individual testing for Covid-19 in the United States, it is of special interest to predict mutation rates in the N region of the genome [16]. High-rate mutations in this region may cause highly undesirable false negatives in the test outcomes. ORF7a encodes for a membrane protein while ORF7b is believed to act as a viral attenuation factor and contributor in human infectivity, similarly to the protein encoded by ORF8. The ORF9b has the role to impede mitochondrial morphology and function and disable the interferon response of the host, while ORF9c appears to block important signaling pathways of the host [22].

### 2.2 Data Acquisition

We used genomes from the GISAID EpiCoV database [24] which contains sequenced viral strains collected from patients across the world. We downloaded the data at three time points in 2020, starting with 04-03-2020, continuing on 04-10-2020 and finishing on 04-14-2020. We revisited the repository on 10-20-2020 to further evaluate the quality of our predictions regarding the mutational supports. At that point of time, 9,271 samples from Asia and more than 30,000 samples from NA and 85,000 samples from Europe were available. For samples made available in April as well as in September, we filtered the datasets only to include nearly-complete samples i.e., those of length $>29,000$>29,000 nts, resulting in a number of samples summarized in Supplementary Table S1, which can be found on the Computer Society Digital Library at http://doi.ieeecomputersociety.org/10.1109/TCBB.2022.3165395. We also downloaded the associated metadata for patient subtyping. Observe again that our method is designed to work with as few samples as possible and is most relevant in the very early stages of a pandemic.

As the first step in our analysis, we used the sequence alignment software MUSCLE [25] to perform pairwise alignment of all the samples with the SARS-CoV-2 sequence of Patient 1, published under the name Wuhan-Hu-1, collected on December 26, 2019 (GenBank accession number MN909847). Furthermore, we also performed PAIRWISE alignment with respect to Patient 1 of two additional continents, Europe and NA. The latter alignment was performed to better determine how the mutational support and mutational distribution depends on a particular geographic context. The choice of Patient 1 is a complex issue. For Asia, Patient 1 is easily identifiable and their utility clear. However, for all other regions there can be several reasonable choices for Patient 1 besides the ones selected based on the timing of sequencing (as the first individual to be sequenced could have arrived from China). For example, one could select a small number of individuals sequenced early on, then cluster all the samples from that month and declare the individual with the largest cluster size as Patient 1. This procedure can be performed for different subregions as well, resulting in more regional Patient 1 choices. We opted for our time-stamped approach as we do not have information about the travel history of local Patient 1s.

We did not make use of majority based rules for SNPs, and did not deal with synonymous/non-synonymous mutations. Also, we did not use multiple sequence alignment (MSA) as we did not try to establish phylogenetic relationships between the various samples. Sequence divergence methods were not considered for the same reason.^1^1.The only phylogeny method very remotely related to our approach is bootstrapping for phylogeny (bootstrap phylogeny [26]). Bootstrapping is used to estimate the confidence of the edges in a phylogenetic tree, which is a very different estimation problem. Note that a similar analysis could have been performed using male/female or old/young Patients 1, but the evidence suggests that the geographic context has a stronger impact on mutational supports of the viral genome [10].

For each aligned pair of samples, we generated a “mutation profile”, a list containing the positions in the reference genome in which the patient aligned to the reference has a substitution mutation. The mutational profile lists are subsequently aggregated over all the patient samples, resulting in a histogram of mutations across all positions in the viral reference genome. The aggregate profiles are further partitioned according to the 11 genes they are located in on the viral genome depicted in Supplementary Fig. S1, available online. The total count of mutations for each location in each gene is used as a sufficient statistics for estimating the mutational support and the distribution of the mutations.

“Spurious” mutations due to sequencing may impact any estimation procedure. Nevertheless, given that it is most likely that sequencing experiments were performed using Next Generation sequencing devices, the error rates are expected to be low, only a fraction of a %. Furthermore, sequencing errors are mostly random, and will hence have very little (if any) influence on the results as they are sporadic. The interested reader is referred to [27] for an analysis of sample errors on the accuracy of small-sample estimators.

To adjust for alignment artifacts introduced by sequencing errors, dropouts and alignment gaps, we removed all gaps encountered in prefixes and suffixes, and sufficiently long gaps ($>10$>10 nts) within the alignments. Most gaps are encountered at the 5’UTR and 3’UTR regions of the genome, as expected from global alignment algorithms.

As there exists a large body of evidence of stratified susceptibility and severity of symptoms across different racial, age and sex groups [28], [29], we performed four different types of mutational support and distribution analyses. In the first set of tests, we split the patient mutation histograms based on sex (male/female), based on age (under 55/over 55) and based on the geographic location (Asia/ NA/ Europe). The age threshold was set by taking into consideration available sample sizes needed for the analysis and the age profile of patients available on GISAID; the threshold also reflects different risk groups for the development of severe symptoms. In addition, we performed the same analysis for a combination of patient features for settings with sufficiently many samples available early in the pandemic, such as males above 55 years of age/females below 55 years of age, from Europe. A partition into different population strata also (partially) mitigates the problem of confounding factors. Note that in all the described cases, “geographic location” refers to the region of infection of the patient and not the region where their sample was sequenced.

Since the number of samples per population type may vary significantly, we performed two tests. In one test we used all samples available, while in another we adjusted for difference in sizes of the sets by subsampling the larger of the two classes to make the sample sets of equal sizes. The number of samples available for various patient subgroups is listed in Supplementary Table S1, available online. For data obtained on 04-03-2020, we used all the samples available for all the classes, without balancing the class sizes. For data retrieved on 04-10-2020 and 04-14-2020, we balanced the classes by subsampling from the larger of the two classes for both age- and sex- based subtypes. For different geographical regions, on 04-10-2020, we used all 615 samples from Asia and subsampled Europe and NA to 1000 samples each. Similarly, we used all 636 samples from Asia and subsampled Europe and NA to 1,774 samples each, for data retrieved on 04-14-2020. By performing the experiments with different sample set sizes one can compare the quality of the estimates obtained using samples from the early stages of a pandemic and those obtained from later stages that are typically much larger.

### 2.3 New Small-Sample Support Estimators

We focus on the *polynomial approximation approach* [30], and significantly improve on it in practice by introducing new weighted Chebyshev polynomial optimization techniques [31]. The weighted approximation method can be seamlessly combined with regularization techniques that use the variance of the estimator in a way that complements features used in ML estimation [32]; and with Semi-Infinite Programming (SIP) solvers that produce the parameters of the estimator. The SIPs can be solved consistently and efficiently through discretization, leading to a Linear Program (LP) of size *decreasing with the number of samples.*

Next, we provide a detailed description of our polynomial estimation method. Recall that the support of a discrete probability distribution is defined as the number of symbols with positive probability of occurrence. We define the mutational support of a virus as the total number of genomic sites mutated in any viral genome in any individual (observed or unobserved due to limited testing), compared to a reference genome. As already pointed out, in our case the reference is the genome of Patient 1, the first sequenced SARS-CoV-2 genome or the genome of regional Patient 1.

The simplest and most commonly used estimator for the support in the large sample regime relies on counting the number of distinct symbols observed. It is well known that these approaches perform poorly for large alphabet sizes (supports) when only a small number of samples from the distribution is available. In this case, they fail to take account for samples that have never been observed due to limited sampling. To see why this is the case, assume that we observe 10 samples from a distribution supported on $\lbrace {1,\ldots,100\rbrace }${1,...,100}. Clearly, with only 10 samples available, our best possible guess for the support size will be the number of distinct symbols observed which is a number $\leq 10$≤10 and far from the correct value 100.

The problem of estimating the support of an unknown probability distribution or estimating the distribution itself in the context of small-sample sets has a long history. The first line of work in this area is attributed to Laplace, who introduced a class of smoothed distribution estimators termed add 1 (or more generally, add constant $c$c estimators). These estimators adjust the counts of observed symbols in order to address the problem of unseen symbols.

Let $P = (p_{1},p_{2},\ldots)$P=(p1,p2,...) be a discrete distribution over some finite alphabet and let $\mathbf{x}^{n}$xn be a vector of $n$n i.i.d samples drawn according to the distribution $P$P. The problem of interest is to estimate the support size, defined as $S(P) = \sum _{i}\mathbf {1}_{\lbrace p_{i}>0\rbrace }$S(P)=∑i1{pi>0}. An important assumption used in our estimation methods is that the minimum non-zero probability of the distribution $P$P is greater than $\frac{1}{k}$1k, for some $k\in \mathbb {R}^{+}$k∈R+, i.e., $\inf \lbrace p\in P\;| \; p>0\rbrace > \frac{1}{k}$inf{p∈P|p>0}>1k. We let $D_{k}$Dk denote the space of all probability distributions satisfying $\inf \lbrace p\in P\; |\; p>0\rbrace > \frac{1}{k}$inf{p∈P|p>0}>1k. A sufficient statistics for $\mathbf{x}^{n}$xn is the empirical distribution (i.e., histogram) $N = (n_{1},n_{2},\ldots),$N=(n1,n2,...), where $n_{i} = \sum _{j=1}^{n}\mathbf {1}_{\lbrace \mathbf{x}_{j}=i\rbrace }$ni=∑j=1n1{xj=i} and $\mathbf {1}_{A}$1A stands for the indicator function of the event $A$A.

To determine the quality of an estimator, we use the most-frequently studied risk model, the minmax risk under normalized squared loss, defined as

\begin{equation*}
R^{\ast }(k,n) = \inf _{\hat{S}}\mathop{\sup} _{P\in D_{k}} \mathbb {E}\left[\left(\frac{\hat{S}(N)-S(P)}{k}\right)^{2}\right]. \tag{1}
\end{equation*}R*(k,n)=infS^supP∈DkES^(N)-S(P)k2.((1))

We seek a support estimator $\hat{S}(N)$S^(N) that minimizes

\begin{align*}
&\mathop{\sup} _{P\in D_{k}} \mathbb {E}\left[\left(\frac{\hat{S}(N)-S(P)}{k}\right)^{2}\right]\tag{2}\\ 
&= \mathop{\sup} _{P\in D_{k}} \left[ \mathbb {E}^{2}\left(\frac{\hat{S}(N)-S(P)}{k}\right)+ var\left(\frac{\hat{S}(N)-S(P)}{k}\right) \right]. \tag{3}
\end{align*}supP∈DkES^(N)-S(P)k2((2))=supP∈DkE2S^(N)-S(P)k+varS^(N)-S(P)k.((3))

The first term within the supremum captures the expected bias of the estimator $\hat{S}(N)$S^(N). The second term represents the variance of the estimator $\hat{S}(N)$S^(N). A“good” estimator should jointly balance out the worst-case contributions of the bias and variance (for the case that only the bias is considered directly, and the variance accommodated for by modifying the bias-optimized solution, the underlying estimator was analyzed in [30]).

To introduce our method, we first describe the class of *polynomial estimators*. Given a positive integer parameter $L$L, we say that an estimator $\hat{S}(N)$S^(N) is a polynomial class estimator with a threshold parameter $L$L (i.e., a $Poly(L)$Poly(L) estimator) if it takes the form $\hat{S}(N) = \sum _{i}g_{L}(n_{i}),$S^(N)=∑igL(ni), where $g_{L}$gL is defined as

\begin{equation*}
g_{L}(j) = \left\lbrace \begin{array}{ll}a_{j} j!+1, &\text{if } j< L\\ 
1, &\text{ otherwise. } \end{array}\right. \tag{4}
\end{equation*}gL(j)=ajj!+1,ifj<L1,otherwise.((4))

The coefficients $a$a satisfy $a_{j} \in \mathbb {R},$aj∈R, and $a_{0} = -1,$a0=-1, (since this choice ensures that $g_{L}(0) = 0$gL(0)=0) and have to be optimized in order to minimize the risk. One can associate an estimator $\hat{S}(N)$S^(N) with its corresponding coefficients $\mathbf {a}$a, i.e.,

\begin{equation*}
Poly(L) = \big\lbrace \mathbf {a}\in \mathbb {R}^{L+1}|a_{0} = -1 \big\rbrace .
\end{equation*}Poly(L)=a∈RL+1|a0=-1.

The authors of [30] proposed using a special form of polynomial estimators in which the coefficients $a_{j}$aj correspond to scaled evaluations of a Chebyshev polynomial of order $L$L. The Chebyshev polynomial of the first kind of degree $L$L is defined as

\begin{equation*}
T_{L}(x) = \cos (L \arccos (x)) = \frac{z^{L}+z^{-L}}{2},
\end{equation*}TL(x)=cos(Larccos(x))=zL+z-L2,

where $z$z is the solution of the quadratic equation $z+z^{-1} = 2x$z+z-1=2x. The polynomial $T_{L}$TL is bounded in the interval $[-1,1]$[-1,1] and may be scaled and shifted to lie in an arbitrary interval $[l,r]$[l,r] based on

\begin{equation*}
R_{L}(x) = -\frac{T_{L}(\frac{2x-r-l}{r-l})}{T_{L}(\frac{-r-l}{r-l})} \triangleq \sum _{j=0}^{L}\tilde{a}_{j}x^{j}.
\end{equation*}RL(x)=-TL(2x-r-lr-l)TL(-r-lr-l)≜∑j=0La ˜jxj.

Clearly, $R_{L}(0) = -1$RL(0)=-1 and $\tilde{a}_{0} = -1$a ˜0=-1.

The Chebyshev polynomial estimator is an estimator for which

\begin{equation*}
\tilde{a}_{j} = \frac{R_{L}^{(j)}(0)}{j!}, \tag{5}
\end{equation*}a ˜j=RL(j)(0)j!,((5))

and it takes the form $\tilde{S}(N)=\sum _{i}\tilde{g}_{L}(n_{i}),$S ˜(N)=∑ig ˜L(ni), where

\begin{align*}
\tilde{g}_{L}(j) = \left\lbrace \begin{array}{ll}\tilde{a}a_{j} j!+1, &\text{ if } j< L,\\ 
1, &\text{ otherwise, } \end{array}\right. \tag{6}\\ 
\text{ with }\;L \triangleq \lfloor c_{0}\log k \rfloor,\; [l,r] \triangleq \left[\frac{n}{k},c_{1} \log k\right].\ 
& \tag{7}
\end{align*}g ˜L(j)=a ˜ajj!+1,ifj<L,1,otherwise,((6))withL≜⌊c0logk⌋,[l,r]≜nk,c1logk.((7))

In [30], the authors suggest setting $c_{0}=0.558$c0=0.558 and $c_{1} = 0.5$c1=0.5 based on their analysis of the bias and variance of the proposed estimator.

The estimator $\tilde{S}(N)$S ˜(N) is order-optimal *in the exponent* under the unbiased risk. Thus, the estimator can be improved by selecting coefficients of $Poly(L)$Poly(L) that jointly optimize the bias and variance term in the risk. We show how to accomplish this task by rewriting the original minmax problem as a regularized exponentially weighted Chebyshev approximation problem [31].

In order to jointly optimize the bias and variance term in the squared loss, we start by directly analyzing $\sup _{P\in D_{k}}\mathbb {E}(\frac{\hat{S}(N)-S(P)}{k})^{2}$supP∈DkE(S^(N)-S(P)k)2. We use the classical Poissonization technique [30] to make the analysis tractable. For a more detailed discussion of the Poissonization process, please refer to the Supplement, available online.

Poissonization arguments lead to

\begin{align*}
&\mathbb {E}\left(\frac{\hat{S}(N)-S(P)}{k}\right)^{2} = \frac{1}{k^{2}}\bigg \lbrace \sum _{i:\lambda _{i}>0}\bigg (\sum _{l=0}^{L}e^{-\lambda _{i}}a_{l}^{2}\lambda _{i}^{l}l!\bigg) \\ 
&+\sum _{i\ne j:\lambda _{i}\lambda _{j}>0}\bigg (e^{-\lambda _{i}}P_{L}(\lambda _{i},\mathbf {a})\bigg)\bigg (e^{-\lambda _{j}}P_{L}(\lambda _{j},\mathbf {a})\bigg)\bigg \rbrace,
\end{align*}ES^(N)-S(P)k2=1k2{∑i:λi>0∑l=0Le-λial2λill!+∑i≠j:λiλj>0e-λiPL(λi,a)e-λjPL(λj,a)},

where $\lambda _{i} \!= \!np_{i}$λi=npi and $P_{L}(\lambda,\mathbf {a}) \!\triangleq \!\sum _{l=0}^{L}a_{l}\lambda ^{l}$PL(λ,a)≜∑l=0Lalλl. Taking the supremum over $D_{k}$Dk we can bound the risk as

\begin{align*}
&\leq \mathop{\sup} _{\mathbf{\lambda}:\lambda _{i}\in [\frac{n}{k}, n]} \frac{1}{k^{2}}\bigg \lbrace \sum _{i:\lambda _{i}>0}\bigg (\sum _{l=0}^{L}e^{-\lambda _{i}}a_{l}^{2}\lambda _{i}^{l}l!\bigg)\\ 
&\quad +\sum _{i\ne j:\lambda _{i}\lambda _{j}>0}\bigg (e^{-\lambda _{i}}P_{L}(\lambda _{i},\mathbf {a})\bigg)\bigg (e^{-\lambda _{j}}P_{L}(\lambda _{j},\mathbf {a})\bigg)\bigg \rbrace \\ 
\end{align*}≤supλ:λi∈[nk,n]1k2{∑i:λi>0∑l=0Le-λial2λill!+∑i≠j:λiλj>0e-λiPL(λi,a)e-λjPL(λj,a)}

which can be further bounded as

\begin{equation*}
\leq \mathop{\sup} _{\lambda \in [\frac{n}{k}, n]}\bigg \lbrace \frac{1}{k}\bigg (\sum _{l=0}^{L}e^{-\lambda }a_{l}^{2}\lambda ^{l}l!\bigg)+\bigg (e^{-\lambda }P_{L}(\lambda,\mathbf {a})\bigg)^{2}\bigg \rbrace .
\end{equation*}≤supλ∈[nk,n]1k∑l=0Le-λal2λll!+e-λPL(λ,a)2.

In the above inequality, we used the Cauchy-Bunyakovsky-Schwarz inequality, the fact that $S(P)\leq k$S(P)≤k and $(\sum _{l=0}^{L}e^{-\lambda }a_{l}^{2}\lambda ^{l}l!)>0,$(∑l=0Le-λal2λll!)>0, for all $\lambda >0$λ>0. Hence, the optimization problem for the coefficients of the polynomial estimator at hand reads as

\begin{equation*}
\inf _{\mathbf {a}\in Poly(L)}\mathop{\sup} _{\lambda \in [\frac{n}{k}, n]}\bigg \lbrace \frac{1}{k}\bigg (\sum _{l=0}^{L}e^{-\lambda }a_{l}^{2}\lambda ^{l}l!\bigg)+\bigg (e^{-\lambda }P_{L}(\lambda,\mathbf {a})\bigg)^{2}\bigg \rbrace . \tag{8}
\end{equation*}infa∈Poly(L)supλ∈[nk,n]1k∑l=0Le-λal2λll!+e-λPL(λ,a)2.((8))

Problem (8) represents an instance of a *regularized weighted Chebyshev approximation problem.* If we ignore the first term in (8), the optimization problem becomes

\begin{equation*}
\inf _{\mathbf {a}\in Poly(L)}\mathop{\sup} _{\lambda \in [\frac{n}{k}, n]}\bigg (e^{-\lambda }P_{L}(\lambda,\mathbf {a})\bigg)^{2}.
\end{equation*}infa∈Poly(L)supλ∈[nk,n]e-λPL(λ,a)2.

The term $e^{-\lambda }P_{L}(\lambda,\mathbf {a})$e-λPL(λ,a) corresponds to the bias of the estimator. It is straightforward to see that the optimal choice of $\mathbf {a}$a for the above problem is a solution to

\begin{equation*}
\inf _{\mathbf {a}\in Poly(L)}\mathop{\sup} _{\lambda \in [\frac{n}{k}, n]}\bigg |e^{-\lambda }P_{L}(\lambda,\mathbf {a})\bigg |. \tag{9}
\end{equation*}infa∈Poly(L)supλ∈[nk,n]|e-λPL(λ,a)|.((9))

The first term $\frac{1}{k} (\sum _{l=0}^{L}e^{-\lambda }a_{l}^{2}\lambda ^{l}l)$1k(∑l=0Le-λal2λll), which corresponds to the variance, may be written as

\begin{align*}
\frac{1}{k}\bigg (\sum _{l=0}^{L}e^{-\lambda }a_{l}^{2}\lambda ^{l}l!\bigg) &= \mathbf {a}^{T}\mathbf {M}(\lambda)\mathbf {a} \triangleq ||\mathbf {a}||_{\mathbf {M}(\lambda)}^{2},\mathbf {M}(\lambda)\\ 
&\triangleq \frac{e^{-\lambda }}{k} Diag(\lambda ^{0}0!,\lambda ^{1}1!,\ldots,\lambda ^{L}L!).
\end{align*}1k∑l=0Le-λal2λll!=aTM(λ)a≜||a||M(λ)2,M(λ)≜e-λkDiag(λ00!,λ11!,...,λLL!).

Clearly, $||.||_{\mathbf {M}(\lambda)}$||.||M(λ) is a valid norm, and consequently, the first term in (8) can be viewed as a regularizer.

Simple algebra reveals that

\begin{align*}
&\mathop{\sup} _{P\in D_{k}}\frac{1}{k}|\mathbb {E}(\hat{S}(N)-S(P))| \leq \mathop{\sup} _{\lambda \in [\frac{n}{k},n]}|e^{-\lambda }P_{L}(\lambda,\mathbf {a})|\tag{10}\\ 
&\leq e^{-\frac{n}{k}}\mathop{\sup} _{\lambda \in [\frac{n}{k},n]}|P_{L}(\lambda,\mathbf {a})|=e^{-\frac{n}{k}}\mathop{\sup} _{\lambda \in [\frac{n}{k},n]}|\sum _{l=0}^{L}a_{l}\lambda ^{l}|. \tag{11}
\end{align*}supP∈Dk1k|E(S^(N)-S(P))|≤supλ∈[nk,n]|e-λPL(λ,a)|((10))≤e-nksupλ∈[nk,n]|PL(λ,a)|=e-nksupλ∈[nk,n]|∑l=0Lalλl|.((11))

where (10) is equivalent to (9), while (11) resembles the problem studied in [30], except for a different optimization interval used within the supremum (the authors of [30] choose a shorter interval in order to decrease the contribution of the variance to the loss). Hence, optimizing (10) should produce an estimator with smaller bias as the exponential weight is inherent to the formulation. The modified bound in (11) is minimized with respect to the coefficients $\mathbf{a}$a, using the minmax property of Chebyshev polynomials [33], [34], resulting in $\tilde{\mathbf{a}}$a ˜.

To solve (8), we more closely examine some results known about weighted Chebyshev approximations [34] and semi-infinite programs. Solving for the problem directly is difficult, so we instead resort to numerically solving the epigraph formulation of problem (8) and proving that the numerical solution is asymptotically consistent.

The epigraph formulation of (8) is of the form

\begin{equation*}
\begin{split}&\mathop{\min} _{t,a_{1},\ldots,a_{L}} t \;\;\;\text{subject to}\\ 
& \bigg \lbrace \frac{1}{k}\bigg (\sum _{l=0}^{L}e^{-\lambda }a_{l}^{2}\lambda ^{l}l!\bigg)+\bigg (e^{-\lambda }P_{L}(\lambda,\mathbf {a})\bigg)^{2}\bigg \rbrace \leq t,\\ 
&\forall \lambda \in [\frac{n}{k}, n], \text{with }a_{0}=-1. \end{split}\tag{12}
\end{equation*}mint,a1,...,aLtsubjectto1k∑l=0Le-λal2λll!+e-λPL(λ,a)2≤t,∀λ∈[nk,n],witha0=-1.((12))

Note that (12) is a semi-infinite programming problem. There are many algorithms that can be used to numerically solve (12), such as the discretization method, and the central cutting plane, KKT reduction and SQP reduction methods [35], [36]. For simplicity, we focus on the discretization method. For this purpose, we first form a grid of the interval $[\frac{n}{k}, n]$[nk,n] involving $s$s points, denoted by $\text{Grid}([\frac{n}{k}, n],s)$Grid([nk,n],s). Problem (12) may consequently be viewed as an LP with infinitely many quadratic constraints, which is not solvable. Hence, instead of solving (12), we focus on the relaxation

\begin{equation*}
\begin{split}&\mathop{\min} _{t,a_{1},\ldots,a_{L}} t \;\;\;\text{subject to}\\ 
& \bigg \lbrace \frac{1}{k}\bigg (\sum _{l=0}^{L}e^{-\lambda }a_{l}^{2}\lambda ^{l}l!\bigg)+\bigg (e^{-\lambda }P_{L}(\lambda,\mathbf {a})\bigg)^{2}\bigg \rbrace \leq t,\\ 
& \forall \lambda \in \text{Grid}([\frac{n}{k}, n],s), \text{with }a_{0}=-1. \end{split}\tag{13} 
\end{equation*}mint,a1,...,aLtsubjectto1k∑l=0Le-λal2λll!+e-λPL(λ,a)2≤t,∀λ∈Grid([nk,n],s),witha0=-1.((13))

The solution of the relaxed problem is asymptotically consistent with the solution of the original problem (i.e., as $s$s goes to infinity, the optimal values of the objectives of the original and relaxed problem are equal). Problem (13) is an LP with a finite number of quadratic constraints that may be solved using standard optimization tools. Unfortunately, the number of constraints scales with the length of the grid interval, which in the case of interest is linear in $n$n. This is an undesired feature of the approach, but it may be mitigated through the following theorem which demonstrates that an optimal solution of the problem may be found over an interval of length proportional to the significantly smaller value $\log \;k$logk, where $\frac{k}{\log \;k} \lesssim n$klogk≲n is the fundamental bound for support estimation. We relegate the proof to the Supplement, available online.

**Theorem 2.1.**

For any $\mathbf {a}\in Poly(L)$a∈Poly(L) and $L=\lfloor c_{0} \; \log \;k \rfloor$L=⌊c0logk⌋, and $c_{0} = 0.558$c0=0.558, let

\begin{equation*}
g(\mathbf {a},\lambda) = \frac{1}{k}\bigg (\sum _{l=0}^{L}e^{-\lambda }a_{l}^{2}\lambda ^{l}l!\bigg)+\bigg (e^{-\lambda }P_{L}(\lambda,\mathbf {a})\bigg)^{2}.
\end{equation*}g(a,λ)=1k∑l=0Le-λal2λll!+e-λPL(λ,a)2.

Then, we have

\begin{align*}
\mathop{\sup} _{\lambda \in [\frac{n}{k}, n]}g(\mathbf {a},\lambda)= \left\lbrace \begin{array}{ll}\mathop{\sup} _{\lambda \in [\frac{n}{k}, 6.5L]}g(\mathbf {a},\lambda) & \text{if }\frac{n}{k}\leq 6.5L\\ 
g(\mathbf {a},\frac{n}{k}) & \text{if }\frac{n}{k}> 6.5L. \end{array}\right.
\end{align*}supλ∈[nk,n]g(a,λ)=supλ∈[nk,6.5L]g(a,λ)ifnk≤6.5Lg(a,nk)ifnk>6.5L.

**Remark**

In weighted approximation theory [31], the problem of bounding the interval over which the supremum is achieved is of significant interest, with many available results. For example, if we ignore the regularization term, we can directly use the Mhaskar-Saff theorem to reduce the length of the interval in the supremum to $\frac{\pi }{2}L$π2L. Our result shows that even when a regularization term is present, we can still restrict the length of the interval to $6.5L$6.5L.

The optimization problem we need to solve to determine our estimator therefore reads as

\begin{equation*}
\begin{split} &\mathop{\min} _{t,a_{1},\ldots,a_{L}} t \;\;\;\text{subject to}\\ 
& \bigg \lbrace \frac{1}{k}\bigg (\sum _{l=0}^{L}e^{-\lambda }a_{l}^{2}\lambda ^{l}l!\bigg)+\bigg (e^{-\lambda }P_{L}(\lambda,\mathbf {a})\bigg)^{2}\bigg \rbrace \leq t,\\ 
&\forall \lambda \in \text{Grid}([\frac{n}{k}, 6.5L],s), \text{with }a_{0}=-1. \end{split}\tag{14}
\end{equation*}mint,a1,...,aLtsubjectto1k∑l=0Le-λal2λll!+e-λPL(λ,a)2≤t,∀λ∈Grid([nk,6.5L],s),witha0=-1.((14))

Since $L=\lfloor c_{0}\; \log \; k \rfloor$L=⌊c0logk⌋, the length of the optimization interval in (14) is proportional to $\log \;k$logk.

It seems intuitive to assume that as $s$s grows, the solution of the relaxed semi-infinite program approaches the optimal solution of the original problem (12). This intuition can be rigorously justified for the case of objective functions and constraints that are “well-behaved”, as defined in [37] and [38]. The first line of work describes the conditions needed for convergence, while the second establishes the convergence rate given that the discretized solver converges. We use these results in conjunction with the properties of our objective SIP to establish the following theorem. The proof is delegated to the Supplement, available online.

**Theorem 2.2.**

Let $s$s be the number of uniformly placed grid points on the interval (14), and let $d\triangleq \frac{6.5L-\frac{n}{k}}{s-1}$d≜6.5L-nks-1 be the length of the discretization interval. As $d \rightarrow 0$d→0, the optimal objective value $t_{d}$td of the discretized SIP (14) converges to the optimal objective value of the original SIP $t^\star$t★. Moreover, the optimal solution is unique $\mathbf {a}^\star$a★. The convergence rate of $t_{d}$td to $t^\star$t★ equals $O(d^{2})$O(d2). If the optimal solution of the SIP is a strict minimum of order one (i.e., if $t-t^\star \geq C||\mathbf {a}-\mathbf {a}^\star ||$t-t★≥C||a-a★|| for some constant $C>0$C>0 and for all feasible neighborhoods of $\mathbf {a}^\star$a★), then the solution of the discretized SIP also converges to an optimal solution with rate $O(d^{2})$O(d2).

**Fig. 1.**
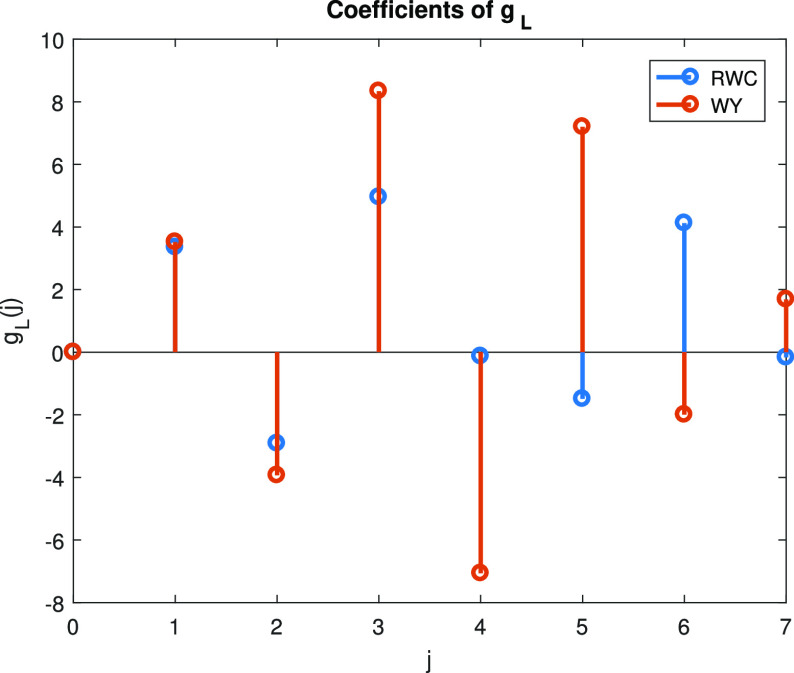
The function $g_{L}$gL for RWC and WY estimator. The parameter setting used for the illustration is $n = k = 10^{6}$n=k=106 and $c_{0} = 0.558$c0=0.558.

In summary, for given parameters $k$k and $n$n, and sample count histograms $N$N we obtain the optimal coefficients of our polynomial estimators by solving the small LP program described above. An example of our polynomial estimator (henceforth termed Regularized Weighted Chebyshev (RWC) estimator) and its scaled coefficients $g_{L}$gL is shown in Fig. 1, along with a corresponding example of a Chebyshev estimator (termed the Wu-Yang (WY) estimator). It is easily observed that the coefficients of the two estimators exhibit very different behaviors: Unlike the Chebyshev case, for which the coefficients have to alternate in sign, our estimators are not constrained to obey this pattern.

The RWC estimators are “additive”: They operate on each symbol separately and the contributions of symbols are linearly combined to obtain the overall support estimate.

We conclude by observing that our RWC estimator can be further heuristically improved in practice by optimizing it with respect to a minmax risk that involves a different scaling factor in the denominator. For this estimator, termed RWC-S, scaling is performed using the unknown support $S(P)$S(P) approximated by the counting support estimator $\hat{S}_{c}(N)$S^c(N)). The analysis of the RWC-S estimator is delegated to the Supplement, available online.

### 2.4 Small-Sample Distribution Estimation

By far the most frequently used method for distribution estimation in the small-sample regime is the Good-Turing estimator [7], which tries to account for the unseen by adjusting the counts of the actually observed symbols. In a slightly modified form the method may be described as follows. For a sequence $\mathbf {x}^{n}$xn of length $n$n over an unknown finite alphabet, we once again let $n_{i}$ni denote the number of times a symbol $i$i appears in $\mathbf {x}^{n}$xn. Furthermore, we let $\varphi _{t}$φt stand for the count of counts, i.e., the number of symbols that appear $t$t times in $\mathbf {x}^{n}$xn. The estimator proposed in [8] combines the Good-Turing and ML estimators, the latter being used for the frequently observed symbols. For symbols that appear $t$t times, if $\varphi _{t+1} > \Omega (t)$φt+1>Ω(t), then the Good-Turing estimate is used to determine the underlying total probability mass, otherwise, the ML estimator is used instead. More precisely, for a symbol appearing $t$t times, if $\varphi _{t+1} > t$φt+1>t we use the Good-Turing estimator, otherwise we use the ML estimator. If $n_{i} = t$ni=t, the estimated probability of the symbol $i$i is computed according to

\begin{equation*}
p_{i} = \left\lbrace \begin{array}{ll}\frac{t}{\eta }, & \text{if t >}{\varphi_{t+1},}\\ 
\frac{\varphi _{t+1}+1}{\varphi _{t}}\;\frac{t+1}{\eta }, &\text{otherwise,} \end{array}\right.
\end{equation*}pi=tη,ift>φt+1,φt+1+1φtt+1η,otherwise,

where $\eta$η is a normalization term that ensures that the obtained values are probability masses. The term $\varphi _{t+1}$φt+1 used in the Good-Turing estimator is replaced by $\varphi _{t+1} + 1$φt+1+1 so that every symbol has a nonzero probability.

The modified Good-Turing estimator is used instead of the classical Good-Turing estimator as the latter poorly estimates the probabilities of high frequency symbols. Modifications of the Good-Turing estimator that take sampling artifacts/errors into account were reported in [27], [39].

The GT method [40] used for comparison first estimates the total probability of seen symbols (e.g., the sample coverage) according to $\hat{C} = 1-\frac{h_{1}}{n}.$C^=1-h1n. Here, $h_{1}$h1 equals the number of different alphabet symbols observed only once in the $n$n samples and $\hat{S}_{\text{c}}(N)$S^c(N) stands for the counting estimator. Vaguely speaking, coverage uses the count of symbols that “were barely seen” (i.e., seen only once) as a proxy for the number of symbols that have not been seen at all. The GT method then computes the support size according to $\hat{S}_{GT}(N) = \frac{\hat{S}_{\text{c}}(N)}{\hat{C}}$S^GT(N)=S^c(N)C^ (see [40]).

We tested our methods against “synthetic” data generated using a multitude of different distributions and the corresponding results are provided in the Supplement, available online. We are not aware of any existing accurate mathematical mutation model for SARS-CoV-2 that can be used to simulate synthetic data [41]. Our methods are also general enough to be applied to other viral genomes provided sufficient number of samples are available along with associated metadata. We discuss Influenza samples in the Supplement, available online, to further illustrate these requirements.

## 3 Results and Discussion

The underlying assumption behind our work is that most of the mutations cannot be observed due to limited testing. The mutational process is inherently nonstationary but that does not preclude an analysis at shorter time scales as changes are gradual. Our studies of the mutational support and mutation distribution are conducted for different patient subpopulations and all ORFs separately in order to determine potential subpopulation differences. Furthermore, we also run our methods at various time points and do a comparative study of the changes in our results over time. The importance of the method lies in the fact that if, due to various biological reasons, there is a substantial change in mutation patterns, our method will be able to capture it with least amount of sampling possible. The proposed estimators are additive, so that estimates for individual genes may be added to obtain the estimate for the whole genome.

First, we observe that by the last small-sample data collection date reported in the manuscript, 04-14-2020, the average number of mutations with respect to the reference was 7.93 (for male patients) and 7.96 (for female patients). This difference is statistically insignificant. For patients older than 55 years, this number was 7.33 while for those younger than 55 the recorded values were substantially higher, amounting to 8.377. For three different continents, Asia, Europe and NA the average number of mutations recorded equaled 13.51, 6.67, and 6.68, respectively. The average number of mutations per patient in Asia is almost twice as large as the corresponding numbers in Europe and NA. A large number of similar sequences from Europe and NA might have contributed to these differences in the mutation frequencies. In all cases, the total number of recorded mutations across all patients is too small to allow for accurate prediction of the actual mutational support using frequency methods. The higher variation in Asian population compared to Europe and NA can also be viewed from a phylogenetic perspective. SARS-CoV-2 has various clades, including S, L, V and G-derived (GR, GV, GS) clades. These clades dominate samples from different regions, which may bias the sampling towards a particular clade. However, this does not impact our estimators and the corresponding results because the estimators only aim to estimate and predict the number of mutations in the population without focusing on particular mutation sites.

Additional statistical tests on the raw number of mutations in each gene are given in the Supplement, available online.

### 3.1 Mutational Support

The first set of results pertains to data collected at a very early stage of the pandemic (04-03-2020) that did not include sufficiently many samples to allow for sample set sizes to be evened out through subsampling. Therefore, for this analysis, all available samples are included, which may create biases due to sample set size differences. The results are listed in Supplementary Tables S6, S7 and S8, available online. They illustrate the difference in the support estimates for two different age groups, sexes and three geographic regions. The nonuniform sample size artifacts do not obscure the most important findings regarding mutation rates in different genes across different age groups, sexes and geographic regions - the same trends persist even when significantly more samples are used in the analysis, as described next. In all the tables, “Counting” refers to the estimator that counts the number of distinct symbols observed, while RWC and RWC-S stand for our proposed two regularized weighted Chebyshev estimators. *Note that whenever the estimate exceeds the length of the gene itself, which is obviously possible as the estimators used can overestimate the true support, we indicated the result by $^\dagger$†. In such cases, one should use the minimum of the length of the gene and the value returned by the estimator.*

Supplementary Tables S9 and S10, available online, list the results for age and sex analogue to those reported for 04-03-2020, obtained using datasets retrieved on 04-10-2020. Table 1 lists corresponding results for geographical regions. The datasets were sufficiently large to allow for random subsampling to obtain equal sample set sizes for all subpopulations considered (excluding Asia).

**TABLE 1:** Support Size for Three Different Geographic Regions Based on 615 Samples From Asia and 1,000 Samples From Europe and NA Each

	Counting			RWC			RWC-S			Gene
Gene	A	E	NA	A	E	NA	A	E	NA	length
ORF1a	827	504	470	1,768	975	948	1,725	919	874	13,203
ORF1b	308	271	244	631	531	478	611	491	432	8,087
S	182	163	142	352	336	269	340	293	243	3,822
ORF3a	91	56	39	174	96	74	168	85	63	828
E	37	12	14	66	21	24	65	21	24	228
M	31	23	17	55	38	28	52	35	27	669
ORF6	3	48	15	3	87	26	3	86	25	186
ORF7a	109	63	51	216	118	94	214	116	93	366
ORF8	340	19	21	335	29	31	339	29	31	366
N	58	72	77	96	121	137	91	108	129	1,260
ORF10	10	26	7	18	48	10	17	48	10	117

The data was retrieved from GISAID on 04-10-2020.

Based on the results of Supplementary Table S9, available online, we see that the mutational supports in populations of different age (cutoff at 55 years) differ substantially for the ORF3a, ORF6 and ORF7a regions. For ORF7a, the older population exhibits almost twice as many mutations compared to the younger population (a difference of $14.8\%$14.8% with respect to the length of the gene), while for ORF6 and ORF3a the corresponding numbers are 1.5 and 1.4, respectively (a difference of $12.4\%$12.4% and $5.7\%$5.7% with respect to the length of the genes, respectively); the estimated mutational supports of the ORF6 and ORF7a regions are close to $1/3$1/3 of the whole gene length for individuals older than 55 years. The mutational differences in the ORF6 and ORF7a region persist with an increase in the number of samples (see the Supplementary Table S11, available online), with an estimated mutational support for the former region equal almost $1/2$1/2 of the gene length. Furthermore, additional differences are observed in the M region which were not apparent when using smaller sample set sizes. The protein encoded by ORF6 was studied in depth during the SARS epidemics [42] and it has been established that the ORF6 protein impairs the nuclear import complex formation (controlling the transport of innate immune regulatory cargo to the nucleus of cells capable of increasing antiviral defenses). The protein encoded by ORF7a has been implicated in inhibiting bone marrow stromal antigen 2 virion tethering [43]. Bone marrow stromal antigen 2, also known as tetherin, is an interferon-induced protein which, when expressed, reduces the release of enveloped viral particles. The significant number of predicted mutations in the ORF7a region of older patients suggests a similar observation as that made for the ORF3a region - a possible effort by the virus to disable or strongly impair the function of the tetherin antigen.

The results pertaining to female/male patients differ substantially from those pertaining to different age groups. The results are listed in Supplementary Table S10, available online, and imply strong differences in the mutation rates of the ORF1b and ORF10 regions. The mutational support of ORF1b in the female population is 1,621 compared to 941 in the male population, which amounts to a $8.4\%$8.4% difference with respect to the length of the ORF. A similar result is true for the ORF10 region, for which no well-understood functions are known. Some recent results suggest, based on different evidence, that ORF10 encodes a functional protein in SARS-CoV-2 and that positive selection is driving the evolution of this region [44].

The above described differences persist with increased sample set sizes. The estimated mutational support for ORF1b is $24\%$24% and $16\%$16% of the length of the region, and for ORF10 $18\%$18% and $32\%$32% of the length of the region, for females and males, respectively (see the Supplementary Table S12, available online). Smaller, yet possibly relevant differences are also observed for the ORF3a and M regions, but these do not persist with increased sample set sizes.

For samples obtained from Asia, Europe and NA the results show that despite the number of samples for Asia being substantially smaller than that from Europe and NA, the predicted mutational support in all regions is higher except for the N and ORF6 genes (with only 3 mutations observed in the ORF6 gene). Particularly for ORF3a and ORF8, the mutation rates are more than 2- and 10-fold higher in Asian patients, respectively (a difference of $10\%$10% and $84\%$84% with respect to the length of the gene, respectively). It is reasonable to assume that these regions are mutated early on in an epidemic and tend to “accumulate” the number of mutations. Also, the substantial differences suggest that the pandemic started *significantly* earlier in Asia than Europe and NA. The ORF3a region is known to encode for a protein that activates the NLRP3 inflammasome [45]. ORF3a proteins are activators of pro-IL-1$\beta$β gene transcription and protein maturation that trigger activation of the NLRP3 inflammasome. The inflammasome has a dual role of boosting the host defense and driving pathologic inflammation. Based on our findings, one possible explanation for the high mutation rate in this region in older populations is that the virus trying to disable the host's immune system and increase its virulence. Recent results show that the ORF8 protein may be acquired from SARS-related coronaviruses present in bats [46], which could explain the large difference in the mutational support through “adaptation” in a human host (for patients in Asia).

Supplementary Tables S11, S12 and S13, available online, show the trends of increase for the mutational support with increased sample sizes. For data collected by 04-14-2020, this includes roughly 9,000 samples. All sample set sizes used are equal (except for Asia, for which the sample set sizes available are substantially smaller), therefore allowing for fair comparisons. Supplementary Table S11, available online, illustrates that when the sample set sizes are equal, no substantial differences are observed in the mutational supports of disparate age groups except in the E, ORF6, ORF7a and ORF8 regions. Given that the difference in the number of mutations in the ORF7a regions persists for several data acquisition dates, the finding appears to be sample-size independent. On the other hand, the substantial differences in the number of mutations in the E region is only evident when sufficiently many samples are available. The E region contains the code for the encapsulation protein of viral RNA, in addition to some spike proteins. In older subjects, this region is subjected to a significantly larger number of mutations than in other groups. This may imply that immunity in elder patient may be dependent on generating antibodies for the encapsulation proteins. Clearly, no conclusive explanation is possible based on limited data sets but the results suggest performing further sampling and analysis for this particular ORF in older patients. Although it has been observed that the immune responses of individuals vary significantly due to the initial viral load, physical health, and the hosts microbiome, no definite link between these features and the mutation rates in the above region can be established due to lack of supporting clinical data at GISAID.

Supplementary Table S12, available online, illustrates surprisingly few differences in the mutational supports of male and female patients once a sufficiently large number of samples is available: Exceptions are the ORF1b and ORF10 regions. For different geographic regions, the most substantial difference observed pertains to the ORF8 region, where samples from Asia exhibit a roughly one order of magnitude larger number of mutations compared to those for samples sequenced in Europe and NA (a difference of $79.2\%$79.2% with respect to the length of the gene). There also exists a marked difference in the mutational support of ORF7a between patients from Europe on one side and patients from Asia and NA on the other (roughly a two-fold difference for Europe and NA).

Ten additional data collection days (starting on 04-03-2020, ending on 04-14-2020) lead to more than twice the samples, and the results for the latter date are shown in Supplementary Fig. S4, available online, along with the standard deviations of the estimators (in order to estimate the variance of an estimator, one needs to subsample the data which requires more samples to start with; hence, the standard deviation is only evaluated for all samples available by 04-14-2020). The additional data samples show that the N region of the SARS-CoV-2 genome exhibits a much more substantial increase in mutations than could have been predicted from early small-set sample sizes, amounting to roughly an average of $23\%$23% of the genome, across populations. This finding is significant as it suggests that genomic regions used as identifiers for the virus may mutate much faster than predicted based on small preliminary sample set information. Nevertheless, the N1 and N2 regions used as primer targets for RT-PCR testing (the use of region N3 as a primer has been discontinued) appear to be largely unmutated. This is illustrated in the Supplementary Table S23, available online, which lists a total of only 8 mutations observed in these regions in the SARS-CoV-2 genomes of US patients. Similar results for mutations in viral genomes of patients from China are presented in Supplementary Table S24, available online.

Table 3 provides results for a finer partition of test samples into two categories, one including males over 55 years of age and another females below 55 years of age, with both populations sampled from Europe. The first category has been empirically observed to be at higher risk of infection and for exhibiting more severe symptoms [29]. Substantial mutational differences are observed in the ORF1b, ORF6 and ORF10 regions. The differences in ORF10 appears to be mostly sex specific, while the age factor may contribute to the differences in the mutation rates of the other two genes. Another important finding is that the mutational support of ORF1b is almost twice as large in the low risk population compared to the high risk population (a difference of $5.9\%$5.9% with respect to the length of the gene). This results may suggest that the large mutational support is a result of a highly competitive virus-host interaction which forces the virus to mutate the proteins encoded by ORF1b in order to gain advantage over the host's immune system.

**TABLE 2:** The p-Values Generated by the Levene Test on the Number of Mutations in Each Gene Based on Data Collected by 14-04-2020

	ORF1a	ORF1b	S	ORF3a	E	M	ORF6	ORF7a	ORF8	N	ORF10
Sex	0.50	0.08	0.94	0.45	0.42	0.64	0.85	0.26	0.51	0.09	0.12
Age	0.11	0.36	0.34	0.70	0.38	0.48	0.43	0.22	0.38	**0.04**	0.42
Region	0.46	0.93	0.75	0.10	0.56	0.27	0.68	0.32	**8e-39**	**6e-16**	**6e-8**

The cases where the null-hypothesis was rejected are shown in bold.

**TABLE 3:** Support Size Differences Between Males $>55$>55 Years and Females $< 55$<55 Years From Europe, Based on 1,078 Samples in Each Group

	Counting		RWC		RWC-S		
	M	F	M	F	M	F	Gene
Gene	$>55$ >55	$< 55$ <55	$>55$ >55	$< 55$ <55	$>55$ >55	$< 55$ <55	length
ORF1a	588	670	1,159	1,374	1,078	1,294	13,203
ORF1b	349	553	686	1,189	638	1,117	8,087
S	209	166	420	329	387	296	3,822
ORF3a	76	61	138	104	124	96	828
E	10	9	17	15	16	14	228
M	27	33	45	58	40	52	669
ORF6	15	28	25	47	24	48	186
ORF7a	31	23	54	36	52	36	366
ORF8	27	28	45	48	43	46	366
N	110	108	197	199	178	183	1,260
ORF10	27	5	28	7	33	7	117

The data was retrieved from GISAID by 04-14-2020.

**Fig. 2.**
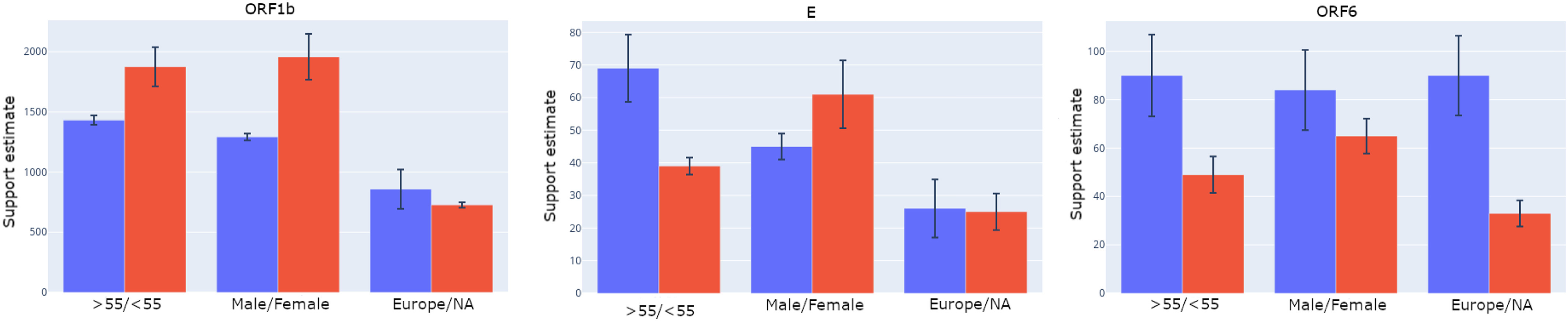
Support sizes for different groups along with their standard deviation estimates. The estimates are based on data collected by 04-14-2020.

Fig. 2 depicts the mutational support sizes, along with the standard deviations of the estimates, for six different patient categories and genes ORF1b, E and ORF6. The corresponding results for the entire set of genes are plotted in Supplementary Fig. S4, available online. The procedure for computing the standard deviations is also described in the Supplement, available online. The mutational supports generated by our procedure have small variances, indicating good concentration of the estimates; some exceptions exist, though and are supported in part by previously observed high rates of mutations in certain SARS-CoV-2 genes [47].

We also performed the Levene test on raw counts of mutations to check for the homogeneity of variance for various subpopulations. The details of the test implementation and the results are available in the Supplement, available online. As one can see from the Table 2, the variances for various subpopulations are equal for all genes except gene N (when stratifying the population according to age) and ORF8, N and ORF10 (when stratifying the population according to regions). We also performed Welch's t-test to check for the equality of means of various subpopulations and the corresponding results are also provided in the Supplement, available online.

We also performed a collection of tests in which alignments and mutational counts were performed with respect to the first sample from the same geographical region. Hence, for patients from Asia, the alignments and mutation counts are still performed with respect to the genome of the Wuhan-Hu-1 patient. For NA, we used the sample USA/WA1/2020 with ID EPI_ISL_404895, while for Europe we used the sample France/IDF0372/2020 with ID EPI_ISL_406596, both being the chronologically first samples from NA and Europe available at GISAID. For this study, we only used samples retrieved by 04-14-2020, and the results are available in Supplementary Tables S14-S17, available online.

It is important to note that for some genes and patient categories it appears the RWC estimates roughly double those of the counting estimator, but this is *not a general trend* of the analysis. For example, the mutational support estimates for ORF8 for male and female are approximately equal to counting estimates (Supplementary Table S10, available online) and more pronounced differences exist across the whole subpopulation spectrum. Similar trends are observed for ORF6 in Asian subjects, and ORF10 across different subpopulations. Furthermore, although *the counting estimates may lead to similar conclusions regarding the trends of mutations in some ORFs, the degree of the trend and the scale of the mutation rates within different regions cannot be fully understood through the use of counting estimates only.* As an illustrative example, the counting estimator implies that there is no difference in the mutational supports of the ORF8 region in young versus old patients (Supplementart Table S9, available online), as the values equal 340 and 341, respectively. On the other hand, the RWC-S estimator predicts mutational supports of 236 and 343, respectively, which show a very different stratification.

We conclude by pointing out that one way to validate the results for our support estimation methods is to compare the results of the counting estimator at a later date with the computed estimates. We compare the mutational supports using the small-sample techniques and the data collected by 04-10-2020 with the actual count generated from data retrieved by 04-14-2020. In this time period, the number of samples increased by roughly 3,000, as may be seen from Supplementary Table S1, available online. The results are listed in Table 4. As may be seen, the estimates obtained based on data acquired by 04-10-2020 for Europe and NA and all open reading frames are excellent matches for the actual counts obtained by 04-14-2020, indicating that the number of samples was sufficient to predict the growth trend. Much more substantial differences are observed for Asia, which can clearly be attributed to the very small sample sizes available from that continent on both 04-10-2020 and 04-14-2020 or potential strong correlations between the mutations in the three aforementioned regions. Other categories that are of interest involve male/female patients for which the actual counts from 04-14-2020 are substantially smaller than the estimates. This is indicative of a large number of potentially unseen mutations harbored by these populations.

**TABLE 4:** RWC-S Estimates for Data Retrieved by 04-10-2020 and the ML Estimates for Data Retrieved by 04-14-2020

Gene	Alg/Date	A	E	NA	$>$> 55	$< $< 55	M	F
ORF1a	RWC-S (04-10)	1,725	919	874	1,896	1,743	2,055	2,175
	ML (04-14)	835	911	804	1,488	1,439	1,478	1,456
ORF1b	RWC-S (04-10)	611	491	432	924	896	941	1,621
	ML (04-14)	316	477	403	787	953	705	991
S	RWC-S (04-10)	340	293	243	458	501	509	519
	ML (04-14)	188	246	209	431	400	405	389
ORF3a	RWC-S (04-10)	168	85	63	171	124	190	158
	ML (04-14)	93	99	81	156	165	169	140
E	RWC-S (04-10)	65	21	24	36	32	36	22
	ML (04-14)	36	15	15	43	26	30	36
M	RWC-S (04-10)	52	35	27	92	77	82	94
	ML (04-14)	31	51	28	79	62	67	69
ORF6	RWC-S (04-10)	3	86	25	64	41	74	50
	ML (04-14)	3	52	21	53	32	50	40
ORF7a	RWC-S (04-10)	214	116	93	103	49	71	84
	ML (04-14)	109	66	135	86	66	68	72
ORF8	RWC-S (04-10)	339	29	31	236	343	344	345
	ML (04-14)	340	32	29	341	343	343	342
N	RWC-S (04-10)	91	108	129	223	265	226	259
	ML (04-14)	60	139	138	201	219	195	204
ORF10	RWC-S (04-10)	17	48	10	39	50	32	19
	ML (04-14)	11	30	10	35	33	31	13

Finally, Table 5 shows the support estimates for samples from patients from Asia for a more recent date of data collection, 10-20-2020. In this case, almost 10,000 samples from Asia are available, which allows one to improve the counting estimators. As may be seen, the differences between the counting estimator and RWC-S values are small. In particular, the ratio of the number of estimated mutations in the ORF E region for the RWC-S and counting method was close to 1.76 in April, and only 1.24 in October. Similar findings hold true for other ORFs.

**TABLE 5:** ML and RWC-S Estimates for Mutational Support Based on 9,271 Samples From Asia Collected by 10-20-2020

	ORF1a	ORF1b	S	ORF3a	E	M	ORF6	ORF7a	ORF8	N	ORF10
ML	5,020	2,691	1,418	464	115	188	90	304	363	510	45
	(77)	(42)	(27)	(19)	(3)	(4)	(3)	(9)	(1)	(6)	(2)
RWC-S	8,481	4,435	2,227	674	143	262	112	333	361	711	61
	(152)	(80)	(61)	(34)	(5)	(8)	(7)	(15)	(7)	(14)	(3)

The standard deviation values are given in parentheses.

### 3.2 Distribution Estimation

**Fig. 3.**
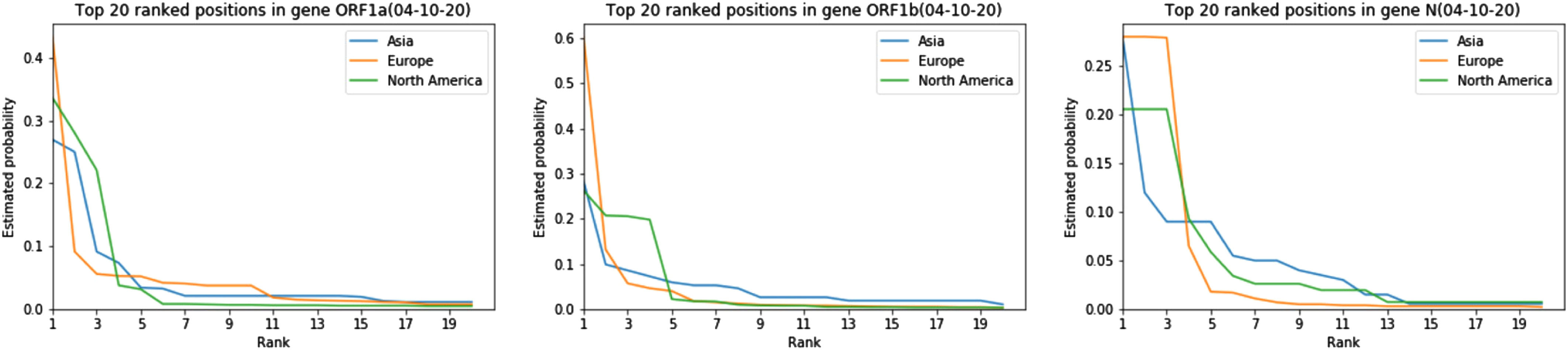
Differences in the estimated distributions of mutations for genes ORF1a, ORF1b and N for different geographic regions based on data collected by 04-10-2020.

Next, we report on the distribution of mutations in the ORF1a,b and N regions of the SARS-CoV-2 virus obtained using the Good-Turing estimator and once again focus on the traits of different subpopulations. We focus on these regions as the first two regions are the longest genes while the N region is of importance for Covid-19 testing in NA. As may be seen from Supplementary Figs. S5 and S6, available online, there is a surprisingly small difference in the distribution of the top-20 mutated sites across different age groups and sexes, except for a marked difference in the largest probability (in particular, in the N region for populations partitioned according to age and populations partitioned according to sex when including larger sample sets from 04-14-2020 as seen in Supplementary Fig. S7, available online). This is especially the case for samples partitioned according to sex, despite the fact that the number of mutations in female subjects in the ORF1b region was close to twice as large as that in male subjects. In addition, the probability of having a mutation at the highest probability sites is substantially larger in “younger” than “older” populations. The trend remains the same for data collected by 04-14-2020 as supported by the results in Supplementary Fig. S7, available online. Supplementary Fig. S8, available online, gives similar results for alignment performed against first sequenced patient from each region. The situation is completely different when comparing the distributions of mutations across different geographic regions (Fig. 3). To compactly summarize the differences in the distributions, we also computed all three pairwise symmetric Kulback-Leibler (KL) divergences for the normalized top-20 mutation probabilities as described below. We also list the Jaccard distances between the sets of 20 most frequent mutations.

**Fig. 4.**
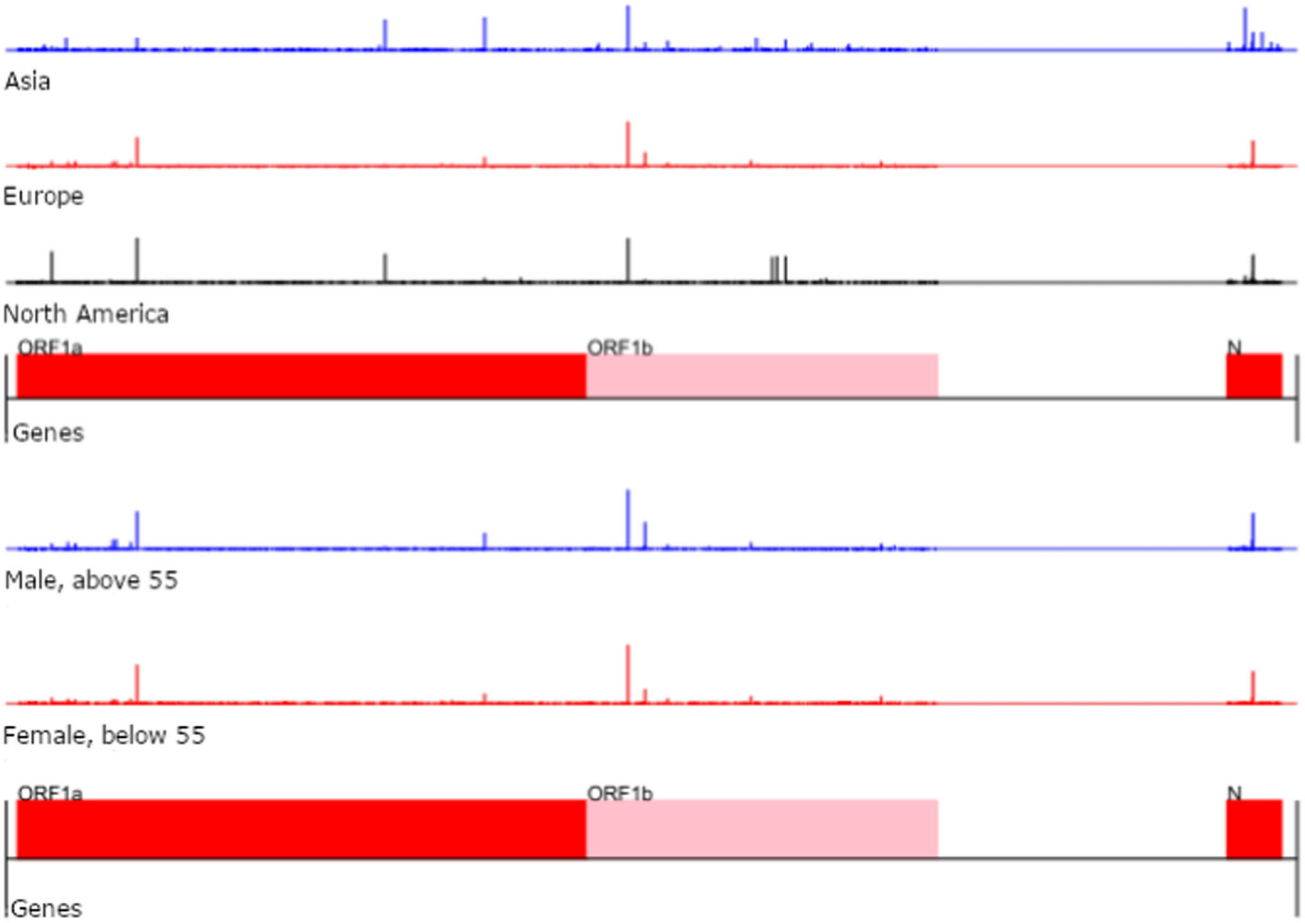
Positions of mutations in the SARS-CoV-2 genome for patients across three different continents, and for European females of age $< 55$<55 and males of age $>55$>55 for data collected by 04-14-2020. The height of the bar is proportional to the estimated probability of mutation.

The distributions of mutations only reveal the statistical landscape of the mutation sites but not their exact locations in the genome. The actual mutated sites in the SARS-CoV-2 genomes are depicted in Fig. 4, in addition to a more detailed set of results presented in the Supplementary Figs. S9 and S10, available online. We selected the latest retrieval data for this analysis as it most accurately reflects the positions undergoing most frequent mutations; we also focused on two cohorts of patients for which the mutational landscapes differ the most. The positional stratification of mutations is substantial for patients from different continents, especially in the N region of the SARS-CoV-2 genome. The largest spread of probability mass is (as expected) observed for patients from Asia which is indicative of the larger exploration rate for mutations in the region where the outbreak originated.

Supplementary Table S18, available online, lists the 10 most frequently mutated sites in the ORF1a region of all previously analyzed patients categories when alignment is performed with respect to the first patient sequenced in the geographic region. For the age and sex groupings, as expected, the top-ten sites are the same except for one difference encountered in both cases. A mutation in position 8,781 of Asian and NA viral samples appears with high frequency but is surprisingly not present in the list of top mutated sites in the European population. Similarly, Supplementary Table S19, available online, lists the 10 most frequently mutated sites in the ORF1b region of all previously analyzed patients categories. As one may expect from the differences in the mutational support, the frequent sites of mutations differ more in this region for different age groups, sexes and continents when compared to the ORF1a region. This is especially the case when viewing the results for different geographic regions as except for the top-ranked site and one more site (i.e., sites 14,407 and 14,804), all other locations are different.

Supplementary Tables S20 and S21, available online, list the top 10 most frequently mutated sites in the ORF3a and ORF8 regions, respectively. One observation pertaining to the top most frequently mutated sites for these genes is related to epstasis in the SARS-CoV-2 genome. In [48], the authors examined 50,000 samples and reported interactions between a total of eight genes, comprising two groups: ORF3a and ORF1ab (in particular, the coding regions for nsp2, nsp6 and nsp12), and ORF8 and ORF1ab (in particular, the coding regions for nsp2, nsp4, nsp13, nsp14). The most frequent epistatic interactions observed include locations 1,059 and 25,563 of the genomes. The probability of mutation for location 1,058 within ORF1a (Supplementary Table S18, available online) is in the top ten positions for all our subpopulations, while 25,562 in ORF3a is in the top two positions across all subpopulations. Note that our locus 1,058 is the same as locus 1,059 and 25,562 is the same as 25,563, the latter numbers being reported in [48]. This shift by one is due to the fact that we used alignment results with respect to a specific reference sequence that has a starting location shifted by one. Similarly, the interaction between 8,781 and 28,143 ranks fifth in [48]. The two aforementioned loci belong to genes ORF1ab and ORF8, respectively. Both positions are ranked high by our distribution estimation method in most of the subpopulations analyzed: The interacting loci found in [48] are those with largest individual marginal probabilities.

Supplementary Table S22, available online, lists top 10 most frequently mutated sites in the N region and suggests significantly fewer stratifications in the mutations of different patient groups. Sex and age does not appear to play a major role, but marked differences are observed in patients from Asia, Europe and NA. Given the large differences in the mutational sites of patients across different continents in the N region it does not come as a surprise that different primers for RT-PCR testing were selected for Asia, Europe and NA. The sites selected for forward and reverse primers by the CDC, i.e., the N1 and N2 region, do not contain a significant number of mutations, as seen from Supplementary Table S23, available online. Mutations are often asymmetric due to polymerase errors and C-to-U substitutions caused host to virus reactions mediated by the APOBEC system [49]: For the N1 and N2 regions, the mutations are predominantly C-to-U substitutions. Similar observations are true for primers selected by China (Supplementary Table S24, available online).

**Fig. 5.**
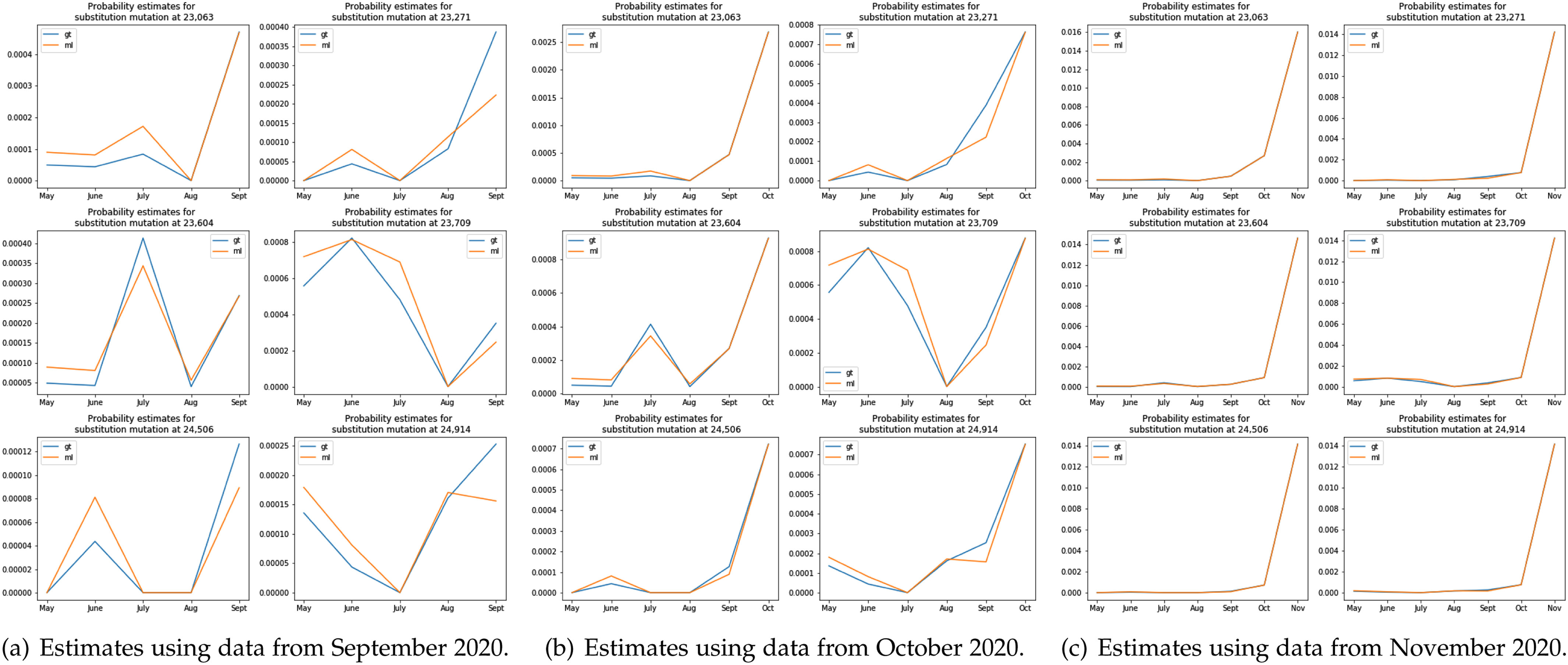
The probability of mutations at six substitution sites in the S region of the UK variant obtained using the GT and ML estimators.

The outlined distribution estimation procedure may also be important in terms of predicting growth trends for certain mutations that may not be easily observed based on frequency counts alone. Fig. 5 demonstrates that the Good-Turing estimator predicted higher probabilities for 4 out of 6 mutation sites in the S region of the UK variant compared to the ML estimator for data collected in September 2020, giving a potentially early indication of the spread of the UK variant (when very few samples with the underlying mutations are seen). Applying GT and ML estimators on data from November 2020 produced identical results.

The symmetric KL divergence between two discrete probability distributions $p$p and $q$q equals

\begin{equation*}
D_{s}(p,q)=D(p||q)+D(q||p),\;\; D(p||q)=\sum _{i}\; p_{i} \log \frac{p_{i}}{q_{i}}.
\end{equation*}Ds(p,q)=D(p||q)+D(q||p),D(p||q)=∑ipilogpiqi.

For the mutation distributions pertaining to Europe-NA, Europe-Asia and Asia-NA, the KL divergences equal 0.672, 0.316 and 0.376 (ORF1a), 0.491, 0.435 and 0.646 (ORF1b), 0.293, 1.021 and 0.303 (N), respectively, for data collected by 04-14-2020. These results indicate that the largest differences in the distributions in the ORF1a region exist between Europe and NA, while the largest differences in the ORF1b region exist between Asia and NA. For the N region, a significant difference between the distributions of mutations is observed between Europe and Asia, and at this point, no simple explanation for this finding is possible. Similarly, the corresponding KL divergences based on the samples collected by 04-10-2020 equal 0.788 (which is substantially larger than the one predicted based on data collected on 04-14-2020), 0.328 and 0.371 (ORF1a), 0.743 (which is substantially larger than the one predicted based on data collected on 04-14-2020), 0.615 and 0.0.755 (ORF1b), 0.315, 0.893 and 0.248 (N), respectively. The results for the KL divergences for the N regions suggest relatively small changes in the distribution of mutations in the N region, and larger changes in the ORF1a and ORF1b regions, which is expected.

Since the previously described distribution estimates do not convey the information about the locations of the most mutated sites but only their frequency, we also list the Jaccard distances of the sets of mutations specific to each tested subpopulation. For two sets $\Sigma _{1}$Σ1 and $\Sigma _{2}$Σ2 over the same ground set $\Sigma$Σ, the Jaccard distance $J(\Sigma _{1},\Sigma _{2})$J(Σ1,Σ2) equals

\begin{equation*}
J(\Sigma _{1},\Sigma _{2})=1-\frac{\Sigma _{1} \cap \Sigma _{2}}{\Sigma _{1} \cup \Sigma _{2}}.
\end{equation*}J(Σ1,Σ2)=1-Σ1∩Σ2Σ1∪Σ2.

As may be seen from Table 6, the largest distances are observed in the E and ORF10 regions, in the first case when comparing patients from Asia and Europe and in the second case when comparing younger female and older males in Europe. The distances in the N region seem to be much smaller, especially between the two categories of patients from Europe. The results for the ORF10 region are rather surprising as they indicate the highest possible difference is observed between males and females on the same continent despite these differences being uniformly small for all other open reading frames. As already pointed out, the function of the ORF10 reading frame is currently unknown but given the marked mutational differences in high-risk and low-risk profiles it is highly likely that this gene plays an important role in guiding disease symptoms. The exact same trends are observed when using alignments with respect to Patient 1 from the underlying geographic region, as listed in Supplementary Table 25, available online.

**TABLE 6:** The Jaccard Distance Between Sets of Mutations From Different Pairs of Geographic Regions, Based on Alignments With Respect to Patient 1 From Wuhan

	ORF1a	ORF1b	S	ORF3a	E	M	ORF6	ORF7a	ORF8	N	ORF10
Asia - Europe	**0.91**	**0.95**	0.91	0.92	**0.98**	**0.92**	**0.96**	*0.74*	0.91	0.86	*0.89*
Europe-NA	0.88	0.89	0.88	0.84	0.89	0.89	*0.86*	**0.91**	0.89	0.82	0.95
Asia - NA	**0.91**	**0.95**	**0.92**	**0.95**	0.91	0.89	**0.96**	0.89	**0.92**	**0.87**	*0.89*
M, $>$>55 - F, $< 55$<55 (Europe)	*0.85*	*0.87*	*0.85*	*0.73*	*0.88*	*0.75*	0.87	*0.83*	*0.83*	*0.77*	**0.97**

Values in *italics* are the smallest in the category, while values in *bold* are the largest.

## 4 Conclusion

We addressed the problem of determining the mutational support and distribution of SARS-Cov-2 in the small-sample regime, of importance during the early stages of an outbreak. To estimate the statistical quantities of interest, we developed a novel, state-of-the-art weighted Chebyshev polynomial estimator for support estimation and adapted the Good-Turing estimator for distribution estimation.

We tested our estimators on various patient subpopulations, including male/female, older/younger and different geographic locations. We observed significant differences in the mutational support of ORF6 and ORF7a between older and younger patients as well as differences in ORF1b and ORF10 between males and females. We also noted that these differences persist when the number of samples increases. We observed that even though the N region of the SARS-CoV-2 genome has a high number of mutations, only a few mutations lay in the primer regions for real time RT-PCR kits recommended by CDC for testing in the USA. Finally, we compared the distributions of mutations among various population groups and computed pairwise symmetric Kulback-Leibler divergences for normalized top-20 mutated positions as well as Jaccard distances for the sets of all mutations for each population.

## Data Availability

The dataset supporting the conclusions of this article is available at the GISAID repository https://www.gisaid.org/. The alignment software and implementations of the RWC and RWC-S algorithms are available at GitHub: https://github.com/rana95vishal/Mutational-landscape-SARS-Cov-2.
